# Molecular Prevalence and Antimicrobial Resistance Profile of *Staphylococcus aureus* and *Staphylococcus pseudintermedius* Isolated From Hospital-Visited Cats

**DOI:** 10.1155/vmi/4879266

**Published:** 2025-08-21

**Authors:** Kazi Shams Al Arefin, Eaftekhar Ahmed Rana, Md Saiful Islam, Belayet Hossain, Md. Ashiqur Rahman, Himel Barua

**Affiliations:** ^1^Department of Microbiology and Veterinary Public Health, Chattogram Veterinary and Animal Sciences University, Khulshi, Chattogram 4225, Bangladesh; ^2^One Health Institute, Chattogram Veterinary and Animal Sciences University, Khulshi, Chattogram 4225, Bangladesh; ^3^Department of Medicine and Surgery, Chattogram Veterinary and Animal Sciences University, Khulshi, Chattogram 4225, Bangladesh

**Keywords:** cats, MDR, MRSA, risk factors, *S. aureus*, *S. pseudintermedius*

## Abstract

Coagulase-positive staphylococci, notably *Staphylococcus aureus* and *Staphylococcus pseudintermedius*, are common pathogens that are frequently associated with suppurative infections in companion animals. This cross-sectional study aimed to investigate the molecular prevalence, antimicrobial resistance profiles, and associated risk factors of *S. aureus* and *S. pseudintermedius* carriage in cats. Standard bacteriological procedures as well as the *nuc* and *pse* genes specific PCR were performed to isolate and identify the *S. aureus* and *S. pseudintermedius*. Methicillin-resistant *S. aureus* (MRSA) and methicillin-resistant *S. pseudintermedius* (MRSP) were identified by detecting the *mec*A gene. A total of 251 swab samples were collected from different body sites of 80 cats. The results showed that 24 (30%) and 5 (6.25%) cats carried *S. aureus* and *S. pseudintermedius*, respectively. *S. aureus* isolates exhibited the highest resistance to penicillin (94.29%) and ampicillin (91.5%), while 60% of *S. pseudintermedius* isolates were resistant to ampicillin, erythromycin, and ciprofloxacin. Thirty-two (91%) *S. aureus* isolates and two (40%) *S. pseudintermedius* isolates demonstrated resistance to ≥ 3 classes of antimicrobials, indicating multidrug resistance (MDR). The prevalence of MRSA was 11.43%, with none of the *S. pseudintermedius* isolates carrying the *mecA* gene and neither *S. aureus* nor *S. pseudintermedius* harboring the *ermA* gene. Multivariable logistic regression identified the previous use of antibiotics as a risk factor for *S. aureus* colonization in cats (odds ratio [OR] 3.84; 95% CI: 1.23–11.95). Cats with otitis externa (OR 46; 95% CI: 3.2–660.4) and steroid use (OR 46; 95% CI: 3.2–660.4) were potential risk factors for *S. pseudintermedius* carriage. The circulation of MDR isolates, including the detection of MRSA, poses a significant concern for the clinical management of companion animals.

## 1. Introduction

Staphylococci represent one of the most important opportunistic bacterial pathogens in humans and animals capable of inflicting a broad diversity of infections. A wide range of *Staphylococcus* species normally inhabit the skin and mucous membranes of the nasal cavity, mouth, and anus in healthy dogs and cats [1]. Based on their pathogenic properties, particularly coagulase production, *Staphylococcus* species are broadly divided into two groups: coagulase-negative staphylococci (CoNS) and coagulase-positive staphylococci (CoPS). CoNS are commonly referred to as commensals and represent one of the major nosocomial pathogens. The majority of the clinical infections are caused by CoPS [2, 3]. Coagulase-positive *Staphylococcus aureus* and *Staphylococcus pseudintermedius* are the most clinically significant staphylococci associated with suppurative infections that can often lead to life-threatening conditions in companion animals [4, 5]. *S. aureus* and *S. pseudintermedius* are common colonizers of various body sites in animals and can lead to a wide range of clinical issues, from minor cutaneous infections to more severe conditions like bacteremia [6–9]. Infections caused by *S. aureus* are particularly prevalent in pet animals with different dermatological conditions [2].

Besides, *S. pseudintermedius* is a part of normal microbiota residing on the skin and mucocutaneous sites of dogs and cats and a notable opportunistic pathogen capable of inflicting a variety of infections, including skin and ear infections and infections of other body tissues and cavities as well as postoperative infections of surgical wounds [10, 11]. Though primarily adapted to dogs, this coagulase-positive bacterium may also infect cats and humans [12, 13]. Due to the simplicity of transmission by contact, aerosols from sneezing and coughing, and saliva, staphylococcal dispersal can be facilitated by an inseparable association between potential hosts. This pathogen has been esteemed as a growing concern to veterinary public health as evidenced by the increasing number of published cases of *S. pseudintermedius* infection in humans [14].

Nowadays, antimicrobial resistance is a great challenge to antimicrobial therapy for animals and humans [15–17]. Numerous recent investigations have shown that staphylococci isolated from cats are resistant to a number of antimicrobial agents; methicillin resistance is of particular concern. The emergence of methicillin-resistant *S. aureus* (MRSA) and methicillin-resistant *S. pseudintermedius* (MRSP) poses a significant problem in veterinary medicine. Methicillin resistance is mediated by the presence of the *mec*A gene encoding production of an altered penicillin-binding protein 2a (PBP2a) that has a low affinity for methicillin and oxacillin and for all beta-lactam antimicrobials [4]. Therefore, methicillin-resistant staphylococci display resistance to other critical antimicrobial classes and consequently limit treatment options. Using veterinary-licensed antimicrobial drugs, clinical treatment of infections with MRSA and MRSP can be challenging and present significant difficulties. Both MRSA and MRSP infections in cats have been reported from various parts of the world [18–20]. It is evident that dogs with significant levels of multidrug resistance and MRSA and MRSP colonization have been the subject of increasing reports [21–24]. In contrast to the information on the magnitude of *S. aureus* and *S. pseudintermedius* infection in dogs and their antimicrobial resistance patterns, little data are available from cats in Bangladesh. This study aimed to figure out the molecular prevalence of *S. aureus* and *S. pseudintermedius* in cats, analyze their antimicrobial resistance patterns, and identify the risk factors associated with MRSA and MRSP colonization in cats.

## 2. Materials and Methods

### 2.1. Ethical Approval and Owner Consent

The entire study process was performed with the approval of the Animal Ethics Committee of Chattogram Veterinary and Animal Sciences University (Approval no. CVASU/Dir (R&E) EC/2023/551-1-11). Additionally, verbal consent was taken from each cat owner prior to sample and data collection within the hospital.

### 2.2. Study Design and Duration

A cross-sectional study was undertaken to determine the prevalence of *S. aureus* and *S. pseudintermedius* in cats visited to SAQTVH, CVASU, for the purpose of general health checkup, treatment, and vaccination during the period between January 2023 and June 2023.

### 2.3. Study Location and Sampling Procedure

Swab samples were collected from the mouth, ear, and perineum of each healthy and sick cat of the hospital-visited cats. One additional swab was also collected from each of the infection sites of a cat that showed skin wounds, dermatitis, abscesses, or ear infections after clinical examination. To collect a sample from a particular body site, a sterile cotton swab was rotated several times in the oral mucosa, the surface of the perineal area, and/or the infection site. Swabs from a body site were placed individually in 5 mL Mueller–Hinton broth (MHB) (HiMedia, India) supplemented with 6.5% NaCl. Following the collection, the samples were immediately shifted to the Department of Microbiology and Veterinary Public Health laboratory, CVASU, for further bacteriological investigation.

### 2.4. Data Collection

A predesigned questionnaire was used to collect the animal demographic and clinical data (cat demographic information such as breed, age, sex, history of management and health status of the cat, and previous history of vaccination and treatment in the cat) from the cat owners by interviewing them (Supporting File 1). Before conducting the study, the questionnaire was pretested on a small group of pet cat owners (not included in the final analysis) to assess relevance, validity, and overall comprehension. A veterinarian was directly involved during the data collection to ensure the accuracy, reliability, and consistency of the responses recorded.

### 2.5. Isolation and Identification of *S. aureus* and *S. pseudintermedius*

The swab samples with 6.5% NaCl-supplemented MHB were placed in an incubator at 37°C and incubated for 24 h. Then, the enriched culture was streaked onto mannitol salt agar (MSA) and incubated at 37°C for 24 h. *S. aureus* and *S. pseudintermedius* were identified on the basis of colony characteristics (smooth, round, and slightly raised yellow colonies) on MSA. Each of the colonies from MSA was streaked onto 5% bovine blood agar and incubated for 24 h at 37°C. Colonies showing the typical appearance of staphylococci on blood agar (pigmented or nonpigmented, round, raised, medium-sized, and hemolytic colonies) were chosen from each plate. Subsequently, primary phenotype identification was performed using Gram's staining, catalase, and tube coagulase tests described by Weese and van Duijkeren [4].

### 2.6. Molecular (PCR) Confirmation of *S. aureus* and *S. pseudintermedius*

Only coagulase-positive isolates were selected for the molecular detection of staphylococcal species by targeting specific *nuc* and *pse* genes for *S. aureus* and *S. pseudintermedius*, respectively [25] (Table 1). Bacterial genomic DNA was extracted using the boiling method described by Rana et al. [28]. Briefly, a loop full of fresh colonies (approximately 3–4 colonies) from a blood agar plate was transferred to an Eppendorf tube containing 100 μL of deionized water. Then, the tubes were vortexed to create a uniform cell suspension. On the top of each tube, a ventilation hole was made to allow extra vapors to escape while the tubes were boiling. The tubes were then placed in a hot water bath and heated at 99°C for 15 min. After boiling, the tubes were placed at −20°C for 5 min. After being frozen, tubes were again placed in a hot water bath at 100°C for 10 min, and the boiled tubes were immediately kept at −20°C for 5 min. The suspension-filled tubes were then centrifuged for 10 min at a speed of 13000 rpm. Supernatants (50 μL) were collected as crude DNA extracts for PCR. A total of 25 μL of the PCR mixture was thermally cycled for initial denaturation at 95°C for 2 min, final denaturation at 95°C for 30 s, annealing at 56°C for 30 s, initial extension at 72°C for 1 min, and final extension at 72°C for 2 min. A total of 30 cycles were run to complete the amplification process. After that, a 1 kb plus DNA ladder (Thermo Scientific, USA) was used to measure the amplicon size on 1.5% agarose gel supplemented with 0.5 μg/mL of ethidium bromide. An ultraviolet chamber was used to visualize the results. The PCR process was monitored using previously confirmed *S. aureus* and *S. pseudintermedius* isolates as positive controls [29] and nuclease-free water as a negative control. All PCR-confirmed *S. aureus* and *S. pseudintermedius* were cultured in 5 mL of brain heart infusion broth (BHIB) (Oxoid Ltd., Basingstoke, UK) and preserved at −80°C with 50% glycerol.

### 2.7. Susceptibility to Antimicrobial Agents

All PCR-confirmed isolates of *S. aureus* and *S. pseudintermedius* were screened for antimicrobial susceptibility testing using the disc diffusion method. The following antimicrobials were used: amoxicillin + clavulanic acid (30 μg), ampicillin (10 μg), cefixime (5 μg), cefoxitin (30 μg), ceftriaxone (30 μg), ciprofloxacin (5 μg), doxycycline (30 μg), erythromycin (15 μg), gentamicin (10 μg), oxacillin (1 μg), penicillin G (10 units), streptomycin (10 μg), sulfamethoxazole–trimethoprim (23.75 + 1.25 μg), tetracycline (30 μg), and vancomycin (30 μg). Based on the Clinical and Laboratory Standards Institute (CLSI) recommendations for veterinary pathogens [30], the zone of inhibition surrounding each disk was measured and interpreted as susceptible (S), intermediate (I), and resistant (R). The zone diameters surrounding the discs of cefoxitin and oxacillin were targeted and interpreted for initial screening of methicillin resistance as described by Schissler et al. [31]. Multidrug resistance (MDR) pattern was identified as *S. aureus* and *S. pseudintermedius* isolates demonstrating resistance against at least three antimicrobial classes described by Magiorakos et al. [32].

### 2.8. Detection of the *mec*A Gene for the Identification of MRSA and MRSP

After the phenotypic screening, only cefoxitin- and oxacillin-resistant isolates were further subjected to the presence of the *mec*A gene by PCR following the procedure described by Larsen et al. [26]. The sequences of oligonucleotide primers used for the detection of the *mec*A gene are shown in Table 1. PCRs were prepared with a 25 μL reaction volume. Previously identified MRSA strain [33] was used as a positive control and nuclease-free water was used as a negative control in each reaction. Finally, the PCR mixture was thermally cycled for initial denaturation at 94°C for 15 min, and 35 cycles were run following final denaturation at 94°C for 30 s, annealing at 59°C for 1 min, initial extension at 72°C for 1 min, and final extension at 72°C for 10 min. An amplified PCR product with 162 bp in 2% agarose gel was confirmed for the presence of the *mec*A gene.

### 2.9. Identification of Erythromycin-Resistant Gene (*erm*A) by PCR

Subsequently, all erythromycin-resistant isolates were screened for the detection of the *erm*A gene described by different authors [27]. The primer sequence of the *erm*A gene is shown in Table 1. Nuclease-free water was used as a negative control, and previously identified *erm*A was used as a positive control [5]. The PCR mixture was 25 μL in volume and thermally cycled for initial denaturation at 93°C for 10 min, final denaturation at 93°C for 1 min, annealing at 52°C for 1 min, initial extension at 72°C for 1 min for 35 cycles, and final extension at 72°C for 5 min. Final PCR products were subjected to gel electrophoresis and visualized using a gel documentation system.

### 2.10. Statistical Analysis

All cats' demographic information and laboratory data were stored in Microsoft Excel 2019 and transferred to STATA Version 16 (StataCorp LP, College Station, Texas, USA) for data summary and descriptive statistics. A cat was considered positive for *S. aureus* and *S. pseudintermedius* when samplings from at least one of the different body sites found target bacteria. Every possible risk factor was analyzed by targeting four findings, such as the existence of *S. aureus*, *S. pseudintermedius*, MRSA, and MRSP. Firstly, a univariable logistic regression model was executed to identify risk factors for the possible outcomes mentioned above. Any variable having a *p* value of ≤ 0.20 was selected for the subsequent multivariable logistic regression model. Finally, variables with a *p* value of ≤ 0.05 were considered significant and were used for the multivariable logistic regression analysis model. A representative heat map was constructed using GraphPad Prism (Version 10.0) to show the distribution of antimicrobial susceptibility patterns of both *S. aureus* and *S. pseudintermedius* isolates.

## 3. Results

### 3.1. Sample Description

A total of 251 swab samples were collected from 80 cats (Table 2). Of which, 48 samples were obtained from 16 apparently healthy cats and 203 swabs from 64 clinically sick cats. Among the total samples, 240 samples came from oral (*n* = 80), ear (*n* = 80), and perineal regions (*n* = 80), and the remaining swabs were from clinical cases of otitis (*n* = 5), wounds (*n* = 4), and dermatitis (*n* = 2) (Table 2).

### 3.2. Carriage of *S. aureus* and *S. pseudintermedius* in Cats

Of the 80 cats, 29 cats (36.25%, 95% CI: 25.78%–47.76%) were positive for CoPS. Among them, 24 cats (30%, 95% CI 20.2%–41.3%) carried *S. aureus* and 5 cats (6.25%, 95% CI 2.06%–13.98%) were positive for *S. pseudintermedius* in at least one body site (Table 2).

### 3.3. Prevalence of Staphylococci, *S. aureus*, and *S. pseudintermedius* in Cats

**Figure 1:**
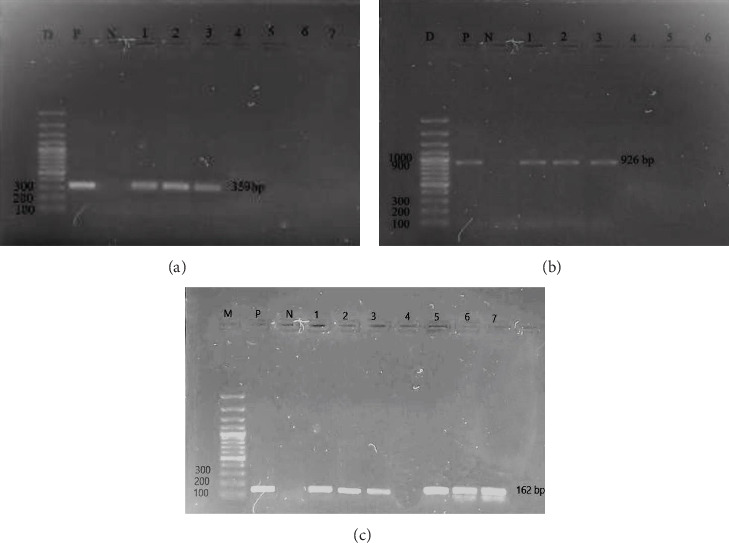
Gel electrophoresis image of PCR products of (a) *S. aureus* (*nuc* gene: 359 bp), (b) *S. pseudintermedius* (*pse* gene: 926 bp), and (c) *mec*A gene: 162 bp) isolates with distinctive amplified bands visible on 1.5% agarose gel. M = 1 kb plus DNA marker, P = positive control, N = negative control, and the subsequent lane showed amplified positive bands.

Out of 251 swab samples, a total of 212 samples (84.46%, CI 79.37%–88.71%) were positive for *Staphylococcus* growth based on the result of morphological appearance and biochemical test (Table 2). Of them, 40 (18.87%, CI 13.83%–24.79%) were coagulase-positive. Among the CoPS, 35 (13.94%, 95% CI: 9.90%–18.85%) and 5 (1.99%, 95% CI: 0.64%–4.58%) were identified as *S. aureus* and *S. pseudintermedius*, respectively, by PCR (Figures 1(a) and 1(b)). The remaining coagulase-negative isolates that were not confirmed at the species level were considered as unclassified staphylococci (*N* = 172). These staphylococci were not investigated further. An overview of the samples collected from different body sites of cats and isolation of *S. aureus* and *S. pseudintermedius* from the samples is shown in Table 2.

### 3.4. Antimicrobial Susceptibility Pattern of *S. aureus*

**Figure 2:**
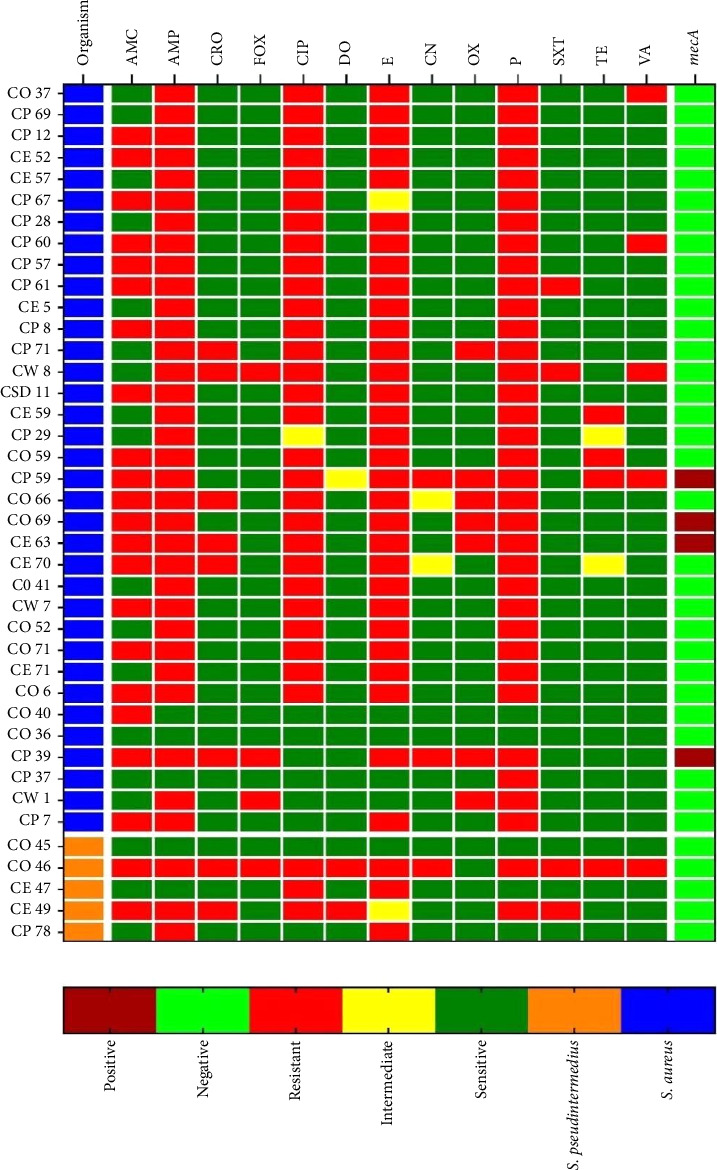
Heat map showing the distribution of the antimicrobial resistance phenotype of *S. aureus* and *S. pseudintermedius* isolates obtained from healthy and clinically sick cats. Each row represents single isolate. Where AMC = amoxicillin + clavulanic acid, AMP = ampicillin, CRO = ceftriaxone, FOX = cefoxitin, CIP = ciprofloxacin, DO = doxycycline, E = erythromycin, CN = gentamicin, OX = oxacillin, P = penicillin, SXT = trimethoprim–sulfamethoxazole, TE = tetracycline, and VA = vancomycin.

All *S. aureus* isolates (*n* = 35) obtained from healthy and sick cats were screened for antimicrobial susceptibility testing and are shown in Table 3 and Figure 2. Highest resistance was observed against penicillin (94.29%), followed by ampicillin (91.5%) and erythromycin (85.71%). Moreover, 80% of *S. aureus* isolates were resistant to ciprofloxacin, and more than 50% of the isolates displayed resistance to amoxicillin–clavulanic acid. Besides, the highest susceptibility was observed against doxycycline, sulfamethoxazole–trimethoprim, and cefoxitin, which were 97.14%, 94.29%, and 91.40%, respectively.

### 3.5. Antimicrobial Susceptibility Pattern of *S. pseudintermedius*

Antimicrobial susceptibility results of *S. pseudintermedius* are shown in Table 3 and Figure 2. Within the isolates, 60% of *S. pseudintermedius* were resistant to ampicillin, erythromycin, and ciprofloxacin. Additionally, 40% of isolates are resistant to trimethoprim–sulfamethoxazole, amoxicillin–clavulanic acid, ceftriaxone, and doxycycline. All isolates showed sensitivity to oxacillin, whereas 20% of the isolates exhibited resistance to cefoxitin phenotypically. Individual antimicrobial resistance profiles of all *S. aureus* and *S. pseudintermedius* isolates are illustrated in a heat map (Figure 2).

### 3.6. MDR Pattern and Detection Rate

The MDR pattern of *S. aureus* (Table 4) and *S. pseudintermedius* is shown in Table 5. A total of 20 different resistance patterns were observed in the case of *S. aureus* isolates (Table 4). The most frequently observed resistance pattern was AMC-AMP-CIP-E-P (*n* = 8), followed by AMP-CIP-E-P (*n* = 7). Of 35 *S. aureus* isolates, 32 (91%) demonstrated multidrug resistance. Four isolates showed resistance to vancomycin. For *S. pseudintermedius*, four different resistance patterns were found (Table 5). Of 5 *S. pseudintermedius* isolates, 2 (40%) isolates were of the MDR pattern, and one isolate showed resistance to vancomycin.

### 3.7. Frequency of *mec*A and *erm*A Genes in *S. aureus* and *S. pseudintermedius*

Frequency of detection of *mec*A genes in *S. aureus* isolates is shown in Table 6. Among the 35 *S. aureus* isolates, 7 isolates (20%; 95% CI: 8.4%–36.94%) were phenotypically resistant to oxacillin, and 3 isolates (8.57%; 95% CI: 1.8%–23%) were phenotypically resistant to cefoxitin. Notably, only 4 isolates (11.43%; 95% CI: 3.20%–26.7%) harbored the *mec*A gene based on PCR results (Figure 1(c)). None of the *S. pseudintermedius* isolates encodes the *mec*A gene.

Although three isolates of both *S. aureus* and *S. pseudintermedius* were phenotypically resistant to erythromycin, none of them carried the *erm*A gene.

### 3.8. Risk Factors Associated With the Presence of *S. aureus* in Cats

The univariable logistic regression analysis has identified five potential risk factors (≤ 0.20) that are associated with the carriage of *S. aureus* in cats (Supporting Table 1). In the subsequent multivariable analysis, only the single variable “previous use of antibiotics” (odds ratio [OR] 3.84; 95% CI: 1.23–11.95, *p* < 0.05) was identified as a significant risk factor associated with the carriage of *S. aureus* (Table 7).

### 3.9. Risk Factors Associated With the Presence of *S. pseudintermedius* in Cats

To identify the risk factors associated with the presence of *S. pseudintermedius*, 15 variables were analyzed using a univariable logistic regression model. Of them, four potential risk factors (≤ 0.20) were retained for subsequent multivariable analysis (Supporting Table 2). Finally, “otitis externa” (OR 46; 95% CI: 3.20–660.44, *p* < 0.05) and the “use of steroids” (OR 46; 95% CI: 3.20–660.44, *p* < 0.05) were identified as significant risk factors associated with the carriage of *S. aureus* (Table 7).

Moreover, only four isolates were positive for MRSA in cats; therefore, no significant association was found among the variables in the logistic regression analysis (Supporting Table 3).

## 4. Discussion

The current study was carried out to investigate the molecular prevalence and antimicrobial resistance profile of *S. aureus* and *S. pseudintermedius* from healthy and clinically sick cats. The possible risks associated with *S. aureus*, *S. pseudintermedius,* and MRSA infections in both clinically healthy and sick cats were also investigated. Cats have been implicated as carriers of both CoPS and CoNS. Among the coagulase-positive staphylococci, the most dominant species are *S. aureus* and *S. pseudintermedius*. Although *S. aureus* and *S. pseudintermedius* were isolated from dogs in Bangladesh before [21], this study is limited to cats in Bangladesh. Since staphylococci are the most predominant commensal infections in cats, a variety of variables, including species, breed, age, sex, management, clinical conditions, and geographic location, may affect prevalence estimates [23,34].

The prevalence of staphylococci in cats does vary in different geolocations. The present study shows that *Staphylococcus* was isolated from 84.46% of specimens taken from various body sites of the cats, which is slightly higher than the study conducted by Bierowiec et al. [35], where 83.40% of samples were positive for staphylococci. But Abraham et al. [36] noticed that CoPS in cats are at 42%, which is higher than that reported in the current investigation. The results of this study showed that CoPS were isolated from 36.25% out of 80 cats. The proportion of coagulase-positive isolates in this study is comparatively lower than that reported in an earlier report from the United States (42%) (Abraham et al. [36]). The overall prevalence of *S. aureus* and *S. pseudintermedius* is 30% and 6.25%, which is lower than that reported in the study conducted by Habibullah et al. [37] at 40.8% and Rana et al. [5] at 12.08%. The higher prevalence of *S. aureus* in cats may indicate frequent contact with humans, contaminated environments, or veterinary settings, whereas the lower prevalence of *S. pseudintermedius* aligns with its primary association with dogs. Moreover, free movements of cats, poor hygiene, and antibiotic selection pressure may further facilitate bacterial colonization and persistence.

Staphylococci were also recovered from different body sites of the cats. The body sites frequently colonized with *S. aureus* and *S. pseudintermedius* are oral and nasal cavity and the perineum along with other sites. In the present study, three body sites were investigated to determine the occurrence of *S. aureus* and *S. pseudintermedius*. The perineal region represented the most frequent colonization site for *S. aureus* (17.5%), followed by the mouth cavity (12.5%). Previously, Bierowiec et al. [23] demonstrated that the highest number of *S. aureus* were isolated from the nares, skin, conjunctival sacs, and anus. Conversely, *S. pseudintermedius* is less frequently colonized in different body sites of cats [5,38]. However, ear swab samples from cats with otitis externa represented the most frequently colonized mucosal site for *S. pseudintermedius*.

The occurrence of *S. aureus* and *S. pseudintermedius* was different in sick and healthy cats. This study demonstrated that healthy cats were carriers of *S. aureus* (12.5%). This proportion was higher in sick cats (34.37%). Similar findings were reported from a study conducted by Abraham et al. [36]. A similar study showed that *Staphylococcus* is more frequently isolated from sick cats than from healthy ones. On the other hand, *S. pseudintermedius* was isolated only from sick cats (7.81%).

In this study, the susceptibility of the isolates was tested against 13 antimicrobials. Both *S. aureus* and *S. pseudintermedius* exhibited great variation in antimicrobial resistance. This diversity of antimicrobial resistance patterns was previously described by different authors, Algammal et al. [39], Perkins et al. [20], and Li et al. [40]. Most of the *S. aureus* isolates in this study showed resistance to penicillin, ampicillin, and erythromycin. Moreover, more than 50% of isolates demonstrated resistance to amoxicillin–clavulanic acid and ciprofloxacin. The organism may have become more resistant to these antimicrobials as a result of repeated exposure to the same antimicrobials and/or lower doses of the medication [21,37]. Additionally, using antimicrobials with similar mechanisms of action against a certain organism may promote the growth of greater resistance [21]. In addition, resistance was observed in *S. pseudintermedius* against ampicillin, erythromycin, and ciprofloxacin, consistent with the results previously reported by different authors [17,23,41,42]. Erythromycin and tetracycline are less commonly used in pet animal practices in Bangladesh, but the resistance against these antimicrobials is alarming for future treatment regimens. However, vancomycin is only prescribed as a last option in human clinics for patients with MRSA infections [43,44].

In the current study, the high prevalence of MDR in 91% (32/35) of *S. aureus* isolates from pet animals like cats raises significant concerns for potential zoonotic transmission as well as antimicrobial stewardship programs. Repeated use of different classes of antimicrobials, such as beta-lactams, macrolides, fluoroquinolones, and tetracyclines, is often prescribed empirically without susceptibility testing that facilitates selection pressure, promoting the survival of resistant strains [21,42]. Moreover, MDR in *S. aureus* and *S. pseudintermedius* could be associated with the harboring of mobile genetic elements such as plasmids, transposons, and integrons, which may carry multiple resistance genes and can be transferred between different bacterial species [45].

Cefoxitin and oxacillin were used as resistant markers for methicillin described by Van Duijkeren et al. [46]. The detection of MRSA in 11.43% (4/35) of *S. aureus* isolates, confirmed by the presence of the *mec*A gene, highlights a significant concern regarding antimicrobial resistance in companion animals. The presence of MRSA in cats suggests possible transmission from pet owners, other pet animals, or the environment, especially in settings with frequent antimicrobial use, such as veterinary clinics. Numerous studies also reported MRSA colonization rates; however, these rates are extremely variable and based on a variety of factors, including geographic region, the animal population investigated, hygienic conditions in the home, and many more [4,47]. MRSA colonization rates were 26.5% in France [19], 30% in Greece [48], and 2% in Malaysia [34]. The prevalence of MRSA was 16.5% and 14% in ear and perineal regions, respectively, whereas Habibullah et al. [37] reported that MRSA in nasal swabs and wound swabs was 30.99% and 64.28% in Bangladesh, respectively. Misic et al. [18] reported that the nares, mouth, and perineum are the major colonization sites in cats. The majority of MRSA isolates were recovered from clinically sick cats rather than from healthy cats, which is comparable to investigations conducted by Lin et al. [49] and Soares Magalhães et al. [50]. Similar studies by Fazal et al. [51] and Rana et al. [52] reporting MRSA in livestock including cattle and goats reinforce the evolutionary source that animals can serve as reservoirs for resistant strains, potentially facilitating cross-species transmission. This finding underscores the public health risk of MRSA, particularly in pet owners and veterinary practitioners. Although one isolate was phenotypically resistant to methicillin, based on the results of PCR, no *mec*A gene was detected in *S. pseudintermedius* isolates. However, a previous study conducted by Rana et al. [5] reported the detection of five MRSP isolates from cats. It may happen due to sampling strategy and differences in the cat population and use of selective antimicrobial pressure. In Poland, one study showed that the prevalence of MRSP in healthy and sick cats was 0.12% and 2.98%, respectively (Bierowiec et al. [23]). Another study conducted in Malaysia by Afshar et al. [34] reported that 2% MRSP was found in pet cats. Notably, in India, a high prevalence (28%) of MRSP was reported in dogs but not in cats [53]. In the United States, Abraham et al. [36] reported that the prevalence of MRSP was 4% in healthy cats, whereas no MRSP was found in cats with inflammatory skin disease. In this investigation, 30 samples of *S. aureus* and three isolates of *S. pseudintermedius* were identified to be resistant to erythromycin; however, the PCR assay revealed that none of these isolates carried the *erm*A gene. The absence of the *erm*A gene suggests that alternative resistance mechanisms may be involved and erythromycin resistance can arise through other *erm* genes (*erm*B, *erm*C, and *erm*F) or efflux pump mechanisms, which could actively expel the antibiotic from bacterial cells. However, Rana et al. [5] reported that 25% of isolates harbored the *erm*A gene in *S. pseudintermedius*. The disparity in carriage rates across research may result from differences in sample size, sample collection methods, cultural methods, health conditions, clinical settings, and management. Moreover, there is a rising issue about an increase in antibiotic resistance in the world. Appropriate management of recurrent infections is essential to reduce antibiotic resistance because bacteria isolated from recurrent infections are more resistant, and resistance is growing over time. The risk of zoonotic transmission should be understood by veterinarians, and appropriate preventative measures should be taken to stop the spread of MRSA and MRSP infections between animals and humans, especially to pet owners.

In the present study, possible risk factors were identified by the univariable and multivariable logistic regression model and showed that cats with wounds accounted for the highest percentage of *S. aureus* isolates (75%), followed by those from otitis externa (20%) and dermatitis (50%), which is highly relevant to the investigation reported by Li et al. [40]. Habibullah et al. [37] reported that the prevalence of *S. aureus* has changed over time as a result of age variation. Previous use of antibiotics is significantly associated with the colonization of *S. aureus* in cats. This factor suggests that antimicrobial exposure may disrupt the normal skin microbiota, reducing bacterial competition and allowing resistant *S. aureus* strains to establish and persist. In addition, multivariable logistic regression showed that cats with otitis externa and use of steroids accounted for the highest percentage of *S. pseudintermedius* carriage (40%). The underlying sick and immunosuppressive health conditions of cats could create favorable conditions for bacterial colonization.

This study has certain limitations. The sample selection was based on registered hospital cases rather than a statistically determined sample size, which may affect the generalizability of the findings. It is important to note that the study population consisted solely of pet cats, with samples collected from a single source, a university teaching veterinary hospital. Therefore, the findings may not fully represent the broader feline population, particularly stray or community cats, which may have different demographic characteristics, antimicrobial exposure, and bacterial prevalence. However, in the context of Bangladesh, stray cats are generally not owned or cared for and survive in natural environments without veterinary intervention. Additionally, capturing these free-roaming cats poses significant logistical and ethical challenges, limiting their inclusion in current studies. Additionally, due to resource constraints, comprehensive genotyping MLST as well as spa typing of the isolates could not be performed. Identifying the sequence types of *S. aureus* and *S. pseudintermedius* circulating in cats would provide valuable epidemiological insights. Future research should address these limitations.

## 5. Conclusions

The carriage rate of CoPS in cats was 36.25%, with *S. aureus* and *S. pseudintermedius* accounting for 13.94% and 1.99%, respectively. Among *S. aureus* isolates, the highest resistance was observed against penicillin (94.29%), followed by ampicillin (91.5%). Additionally, 60% of *S. pseudintermedius* isolates were resistant to ampicillin, erythromycin, and ciprofloxacin. Notably, 91% of *S. aureus* and 40% of *S. pseudintermedius* isolates were identified as MDR. Moreover, 11.43% of *S. aureus* isolates carried the *mecA* gene, confirming them as MRSA. The risk of colonization with *S. aureus* and *S. pseudintermedius* was significantly higher in sick cats than in healthy ones. Therefore, strengthening One Health approaches, integrating veterinary and human medicine, and enforcing antibiotic stewardship policies are critical to mitigating the MDR issues and preventing the cross-species transmission of resistant pathogens between animals and humans.

## Figures and Tables

**Table 1:** Primer sequences used for the identification of species and resistance gene amplification of *S. aureus* and *S. pseudintermedius*.

Species	Target gene	Primer designation	Oligonucleotide primer 5′–3′	Annealing temperature	Amplicon size (bp)	References
Species identification	*nuc*	*nuc*-F3	TCGCTTGCTATGATTGTGG	56°C	359	Sasaki et al. [25]
		*nuc*-R	GCCAATGTTCTACCATAGC			
	*pse*	*Pse*-F2	TRGGCAGTAGGATTCGTTAA	56°C	926	Sasaki et al. [25]
		*Pse*-R5	CTTTTGTGCTYCMTTTTGG			

Resistant genes	*mec*A	*mec*A forward	TCCAGATTACAACTTCACCAGG	59°C	162	Larsen et al. [26]
		*mec*A reverse	CCACTTCATATCTTGTAACG			
	*erm*A	*erm*A forward	TCTAAAAAGCATGTAAAAGAA	52°C	645	Shekarabi et al. [27]
		*erm*A reverse	CTTCGATAGTTTATTAATATTAG			

**Table 2:** Distribution of coagulase-positive *S. aureus* and *S. pseudintermedius* in different body sites of cats.

Sources	No. of samples	No. of coagulase-positive *Staphylococcus* sp. (%; 95% CI)	No. of *S. aureus* (%; 95% CI)	No. of *S. pseudintermedius* (%; 95% CI)
Oral	80	11 (13.75; 7.07–23.27)	10 (12.5; 6.16–21.78)	1 (1.25; 0.03–6.76)
Perineal	80	15 (18.75; 10.89–29.03)	14 (17.5; 9.91–27.61)	1 (1.25; 0.03–6.76)
Ear	80	7 (8.75; 3.59–17.20)	6 (7.5; 2.80–15.61)	1 (1.25; 0.03–6.76)
Otitis externa	5	3 (60; 14.66–94.72)	1 (20; 0.50–71.64)	2 (40; 5.27–85.33)
Dermatitis	2	1 (50; 1.26–98.74)	1 (50; 1.26–98.74)	0
Wound	4	3 (75; 19.41–99.37)	3 (75; 19.41–99.37)	0
Total	251	40 (15.93; 11.63–21.06)	35 (13.94; 9.90–18.85)	5 (1.99; 0.64–4.58)

Abbreviation: CI = confidence interval.

**Table 3:** Antimicrobial susceptibility pattern of *S. aureus* and *S. pseudintermedius* isolated form cats.

Organism	Susceptibility pattern	AMC	AMP	CRO	FOX	CIP	DO	E	CN	OX	P	SXT	TE	VA
*S. aureus* (35)	Sensitive (%)	15 (42.86)	3 (8.57)	29 (82.86)	32 (91.43)	6 (17.14)	34 (97.14)	4 (11.43)	31 (88.57)	28 (80)	2 (5.71)	33 (94.29)	30 (85.71)	31 (88.57)
	Intermediate (%)	0	0	0	0	1 (2.86)	1 (2.86)	1 (2.86)	2 (5.71)	0	0	0	2 (5.71)	0
	Resistant (%)	20 (57.14)	32 (91.43)	6 (17.14)	3 (8.57)	28 (80)	0	30 (85.71)	2 (5.71)	7 (20)	33 (94.29)	2 (5.71)	3 (8.57)	4 (11.43)

*S. pseudintermedius* (5)	Sensitive (%)	3 (60)	2 (40)	3 (60)	4 (80)	2 (40)	3 (60)	1 (20)	4 (80)	5 (100)	3 (60)	3 (60)	4 (80)	4 (80)
	Intermediate (%)	0	0	0	0	0	0	1 (20)	0	0	0	0	0	0
	Resistant (%)	2 (40)	3 (60)	2 (40)	1 (20)	3 (60)	2 (40)	3 (60)	1 (20)	0	2 (40)	2 (40)	1 (20)	1 (20)

*Note:* AMC = amoxicillin + clavulanic acid, AMP = ampicillin, CRO = ceftriaxone, FOX = cefoxitin, CIP = ciprofloxacin, DO = doxycycline, E = erythromycin, CN = gentamicin, OX = oxacillin, P = penicillin, SXT = trimethoprim–sulfamethoxazole, TE = tetracycline, VA = vancomycin.

**Table 4:** Antimicrobial resistance pattern of *S. aureus* isolated from cats.

Sl. no.	Phenotypic resistance pattern	No. of isolates displaying resistance
1	AMP-CIP-E-P-VA	1
2	AMP-CIP-E-P	7
3	AMC-AMP-CIP-E-P	8
4	AMC-AMP-CIP-P	1
5	AMC-AMP-CIP-E-P-VA	1
6	AMC-AMP-CIP-E-P-SXT	1
7	AMP-CRO-CIP-E-OX-P	1
8	AMP-CRO-FOX-CIP-E-P-SXT-VA	1
9	AMP-CIP-E-P-TE	1
10	AMP-E-P	1
11	AMC-AMP-CIP-E-P-TE	1
12	AMC-AMP-CIP-E-CN-OX-P-TE-VA	1
13	AMC-AMP-CRO-CIP-E-OX-P	2
14	AMC-AMP-CIP-E-OX-P	1
15	AMC-AMP-CRO-CIP-E-P	1
16	AMC-AMP-CRO-FOX-E-CN-OX-P	1
17	P	1
18	AMP-FOX-OX-P	1
19	AMC-AMP-E-P	1
20	AMC	1

*Note:* AMC = amoxicillin + clavulanic acid, AMP = ampicillin, CRO = ceftriaxone, FOX = cefoxitin, CIP = ciprofloxacin, DO = doxycycline, E = erythromycin, CN = gentamicin, OX = oxacillin, P = penicillin, SXT = trimethoprim–sulfamethoxazole, TE = tetracycline, VA = vancomycin.

**Table 5:** Antimicrobial resistance pattern of *S. pseudintermedius* isolated from cats.

Sl. no.	Phenotypic resistance pattern	No. of isolates displaying resistance
1	AMC-AMP-CRO-FOX-CIP-DO-E-CN-P-SXT-T-VA	1
2	CIP-E	1
3	AMC-AMP-CRO-CIP-DO-P-SXT	1
4	AMP-E	1

**Table 6:** Phenotypic and molecular detection of methicillin-resistant *S. aureus* isolates.

Source of isolates	No. of *S. aureus* isolates	Oxacillin-resistant *S. aureus* isolates	Cefoxitin-resistant *S. aureus* isolates	*mec*A-positive isolates	Prevalence (95% CI)
Oral	10	2	0	1	1 (0.25–44.5)
Perineal	14	3	1	2	14.3 (1.78–42.8)
Ear	6	1	0	1	16.67 (0.42–64.12)
Otitis externa	1	0	0	0	—
Wound	3	1	2	0	—
Dermatitis	1	0	0	0	—
Total	35	7	3	4	11.43 (3.20–26.7)

**Table 7:** Multivariable logistic regression for determining the risk factors independently associated with *S. aureus* and *S. pseudintermedius* from multiple body sites of cats.

Outcome variable	Explanatory variable	Description	Odds ratio (OR) (95% CI)	*p* value
*S. aureus*	Previous use of antibiotics	Yes	3.84 (1.23–11.95)	^∗^0.020
		No	1	Reference

*S. pseudintermedius*	Otitis externa	Yes	46 (3.20–660.44)	^∗^0.005
		No	1	Reference
	Use of steroids	Yes	46 (3.20–660.44)	^∗^0.005
		No	1	Reference

*Note:* significance ^∗^(≤0.05).

Abbreviation: CI, confidence interval.

## Data Availability

The data that represent the findings of this study are available within the article.

## Supporting Information

Supporting Information 1 Supporting file 1: Questionnaire.

Supporting Information 2 Supporting Table 1: Univariable logistic regression analysis to identify the risk factors for the carriage of *S. aureus* in cats.

Supporting Information 3 Supporting Table 2: Univariable logistic regression analysis to identify the risk factors for the carriage of *S. pseudintermedius* in cats.

Supporting Information 4 Supporting Table 3: Univariable logistic regression analysis to identify the risk factors for the presence of MRSA in cats.
