# Supplementary material for: Molecular Prevalence and Antimicrobial Resistance Profile of Staphylococcus aureus and Staphylococcus pseudintermedius Isolated From Hospital-Visited Cats
Source: Vet Med Int. 2025 Aug 21;2025:4879266. doi: 10.1155/vmi/4879266 (PMC12393940; doi:10.1155/vmi/4879266)
Supplement: Supporting Information 1 — Supporting file 1: Questionnaire. [file 4879266.f1.docx]

**Title: Molecular prevalence and antimicrobial resistance profile of S*taphylococcus aureus* and *Staphylococcus pseudintermedius* isolated from hospital visited cats**

**Sample ID:**

1. **Cat Demographic Information**
   1. Breed
   2. Age:
   3. Sex:
2. **History of Management:**
   1. Is the Cat showered every day? Yes/No
   2. Residence of the Cat: Inside the house/Outside the house
   3. Bedding with the cat? Yes/No
   4. Use of any disinfectants? Yes/No
3. **Health Status of Cat:**
   1. Is the cat healthy? Yes/No
   2. Any dermatitis or skin lesion present? Yes/No
   3. Any wound in skin? Yes/No
   4. Presence of otitis externa? Yes/No
   5. Presence of any oral lesion? Yes/No
   6. Presence of pneumonia? Yes/No
   7. Other disease conditions:
4. **History of Vaccination and Treatment in Cat:**
   1. Vaccination: Yes/No
   2. Deworming: Yes/No
   3. Previous use of any antibiotic? Yes/No
   4. Present use of any antibiotic? Yes/No
   5. Use of any topical cream? Yes/No
   6. Use of any steroidal drugs? Yes/No
