# Supplementary material for: Molecular Prevalence and Antimicrobial Resistance Profile of Staphylococcus aureus and Staphylococcus pseudintermedius Isolated From Hospital-Visited Cats
Source: Vet Med Int. 2025 Aug 21;2025:4879266. doi: 10.1155/vmi/4879266 (PMC12393940; doi:10.1155/vmi/4879266)
Supplement: Supporting Information 2 — Supporting Table 1: Univariable logistic regression analysis to identify the risk factors for the carriage of S. aureus in cats. [file 4879266.f2.docx]

| **Variables** | **Co-**  **variables** | **No. of cats** | **No. of cat’s positive for**  ***S. aureus* (%)** | **95% CI** | **OR (95%CI)** | ***p*-**  **value** |
| --- | --- | --- | --- | --- | --- | --- |
| Breed | Bengal Cat | 38 | 10 (26.31) | 13.40 - 43.10 | Reference | 0.493 |
|  | Persian | 42 | 14 (33.33) | 19.56 - 49.55 | 1.4 (0.53 - 3.67 |  |
| Age | Kitten | 18 | 5 (27.77) | 9.69 - 53.48 | Reference | 0.634 |
|  | Young | 44 | 15 (34.01) | 20.49 - 49.51 | 1.34 (0.40 - 4.4) |  |
|  | Adult | 18 | 4 (22.22) | 6.41 - 47.64 | 0.74 (0.16 - 3.38) |  |
| Sex | Male | 56 | 16 (28.57) | 17.29 - 42.21 | Reference | 0.671 |
|  | Female | 24 | 8 (33.3) | 15.63 - 55.32 | 1.25 (0.45 - 3.49 |  |
| Status of  Health | Sick | 64 | 22 (34.37) | 22.94 - 47.30 | Reference | 0.088* |
|  | Healthy | 16 | 2 (12.5) | 1.55 - 38.34 | 0.27 (0.056 - 1.30) |  |
| Use of Disinfectant | No | 7 | 2 (28.57) | 3.67 - 70.95 | Reference | 0.931 |
|  | Yes | 73 | 22 (30.13) | 19.94 - 42.01 | 1.07 (0.19 - 5.98) |  |
| Dermatitis | Not Found | 78 | 23 (29.48) | 19.69 - 40.89 | Reference | 0.532 |
|  | Found | 2 | 1 (50) | 1.25 - 98.74 | 2.3 (0.14 - 39.8) |  |
| Wound | No | 76 | 21 (27.63) | 17.98 - 39.08 | Reference | 0.044^*^ |
|  | Yes | 4 | 3 (75) | 19.41 - 99.36 | 7.85 (0.77 - 79.8) |  |
| Otitis externa | No | 75 | 23 (30.66) | 20.53 - 42.38 | Reference | 0.614 |
|  | Yes | 5 | 1 (20) | 0.51 - 71.64 | 0.56 (0.05 - 5.3) |  |
| Shower interval | Irregular | 64 | 18 (28.12) | 17.59 - 40.76 | Reference | 0.471 |
|  | Regular | 16 | 6 (37.5) | 15.19 - 64.56 | 1.53 (0.485 - 4.84) |  |
| Vaccination | No | 40 | 16 (40) | 24.86 - 56.67 | Reference | 0.055* |
|  | Yes | 40 | 8 (20) | 9.05 - 35.64 | 0.375 (0.137 - 1.02) |  |
| Deworming | No | 36 | 14 (38.89) | 23.14-56.53 | Reference | 0.116* |
|  | Yes | 44 | 10 (22.72) | 11.47 - 37.84 | 0.462 (0.174 - 1.22) |  |
| Previous use of antibiotics | Yes | 45 | 19 (42.22) | 27.65 - 57.84 | 4.38 (1.43 - 13.38) | 0.009^*^ |
|  | No | 35 | 5 (14.28) | 4.80 - 30.25 | Reference |  |
| Present use  of antibiotics | Yes | 17 | 6 (35.29) | 14.20 - 61.67 | 1.36 (0.438 - 4.24) | 0.595 |
|  | No | 63 | 18 (28.57) | 17.89 - 41.34 | Reference |  |
| Use of  Steroid | Yes | 5 | 2 (40) | 5.27 - 85.33 | 1.60 (0.25 - 10.28 | 0.614 |
|  | No | 75 | 22 (29.33) | 19.38 - 40.97 | Reference |  |
| Use of Topical  Cream | Yes | 6 | 2 (33.3) | 4.32 - 77.72 | 1.18 (0.201 - 6.93) | 0.854 |
|  | No | 74 | 22 (29.72) | 19.65 - 41.48 | Reference |  |

**Supplementary table 1**: Univariable logistic regression of analysis to identify the risk factors for the carriage of *S. aureus* in cats.
