# Supplementary material for: Molecular Prevalence and Antimicrobial Resistance Profile of Staphylococcus aureus and Staphylococcus pseudintermedius Isolated From Hospital-Visited Cats
Source: Vet Med Int. 2025 Aug 21;2025:4879266. doi: 10.1155/vmi/4879266 (PMC12393940; doi:10.1155/vmi/4879266)
Supplement: Supporting Information 3 — Supporting Table 2: Univariable logistic regression analysis to identify the risk factors for the carriage of S. pseudintermedius in cats. [file 4879266.f3.docx]

**Supplementary table 2**: Univariable logistic regression analysis to identify the risk factors for the carriage of *S. pseudintermedius* in cats.

| **Variables** | **Co-**  **variables** | **No. of**  **Cats** | **No. of cat’s positive for**  ***S*. *pseudintermedius*** | **95% CI** | **OR (95%CI)** | ***p*-**  **value** |
| --- | --- | --- | --- | --- | --- | --- |
| Breed | Bengal Cat | 38 | 3 (7.89) | 1.65 - 21.4 | Reference | 0.562 |
|  | Persian | 42 | 2 (4.76) | 0.58 - 16.16 | 0.583 (0.09 - 3.69) |  |
| Age | Kitten | 18 | 2 (11.11) | 1.38 - 34.71 | Reference | 1.000 |
|  | Young | 44 | 1 (2.27) | 0.06 - 12.02 | 0.18 (0.01 - 2.19) |  |
|  | Adult | 18 | 2 (11.11) | 1.38 - 34.71 | 1 (0.12 - 7.99) |  |
| Sex | Male | 56 | 4 (7.14) | 1.98 -17.29 | Reference | 0.601 |
|  | Female | 24 | 1 (4.16) | 0.105 - 21.12 | 0.565 (0.0598 - 5.33 |  |
| Status of  Health | Healthy | 16 | 0 | - | 1 (omitted) | 0.25 |
|  | Sick | 64 | 5 (7.81) | 2.58 - 17.29 | Reference |  |
| Use of Disinfectant | Yes | 73 | 3 (4.10) | 0.85 - 11.54 | 0.107 (0.014 - 0.796) | 0.045* |
|  | No | 7 | 2 (28.57) | 3.66 - 70.95 | Reference |  |
| Dermatitis | Found | 2 | 0 | - | 1 (omitted) | 0.712 |
|  | Not Found | 78 | 5 (6.41) | 2.11-14.32 | Reference |  |
| Wound | Yes | 4 | 0 | - | 1 (omitted) | 0.596 |
|  | No | 76 | 5 (6.57) | 2.17 - 14.68 | Reference |  |
| Otitis externa | Yes | 5 | 2 (40) | 0.63 - 80.58 | 16(1.9 - 134.57) | 0.001^*^ |
|  | No | 75 | 3 (4) | 0.83-11.24 | Reference |  |
| Shower  interval | Regular | 16 | 0 | - | 1 (omitted) | 0.25 |
|  | Irregular | 64 | 5 (7.8) | 2.58 - 17.29 | Reference |  |
| Vaccination | Yes | 40 | 3 (7.5) | 1.57 - 20.38 | 1.54 (0.24 - 9.75) | 0.643 |
|  | No | 40 | 2 (5) | 0.61 - 16.91 | Reference |  |
| Deworming | Yes | 44 | 3 (6.81) | 1.42 - 18.65 | 1.2 (0.19 - 7.87) | 0.815 |
|  | No | 36 | 2 (5.55) | 0.68 - 18.66 | Reference |  |
| Previous use  of antibiotics | Yes | 45 | 1 (2.22) | 0.05 - 11.77 | 0.176 (0.018 - 1.65) | 0.09* |
|  | No | 35 | 4 (11.42) | 3.20 - 26.73 | Reference |  |
| Present use of  antibiotics | Yes | 17 | 1 (5.88) | 0.15 - 28.68 | 0.92 (0.096 - 8.83) | 0.943 |
|  | No | 63 | 4 (6.34) | 1.76 - 15.46 | Reference |  |
| Use of Steroid | Yes | 5 | 2 (40) | 0.63 - 80.58 | 16 (1.9 -134.57) | 0.001^*^ |
|  | No | 75 | 3 (4) | 0.83 - 11.24 | Reference |  |
| Use of Topical  Cream | Yes | 6 | 0 | - | 1 (omitted) | 0.511 |
|  | No | 74 | 5(6.75) | 2.23 - 15.0 | Reference |  |

Abbreviation: OR= odds ratio, CI= confidence interval, *≤0.05=significance
