# Supplementary material for: Molecular Prevalence and Antimicrobial Resistance Profile of Staphylococcus aureus and Staphylococcus pseudintermedius Isolated From Hospital-Visited Cats
Source: Vet Med Int. 2025 Aug 21;2025:4879266. doi: 10.1155/vmi/4879266 (PMC12393940; doi:10.1155/vmi/4879266)
Supplement: Supporting Information 4 — Supporting Table 3: Univariable logistic regression analysis to identify the risk factors for the presence of MRSA in cats. [file 4879266.f4.docx]

**Supplementary table 3**: Univariable logistic regression of analysis to identify the risk factors for the presence of MRSA in cats.

| **Variables** | **Co-**  **variables** | **No. of cats** | **No. of cat’s positive for**  **MRSA (%)** | **95% CI** | **OR (95%CI)** | **p-**  **value** |
| --- | --- | --- | --- | --- | --- | --- |
| Breed | Bengal Cat | 38 | 1 (2.63) | 0.06 - 13.8 | Reference | 0.343 |
|  | Persian | 42 | 3 (7.14) | 1.49 - 19.48 | 2.84 (0.28 - 28.5) |  |
| Age | Kitten | 18 | 1 (5.5) | 0.14 - 27.29 | Reference | 1.000 |
|  | Young | 44 | 2 (4.5) | 0.55 - 15.47 | 0.80 (0.068 - 9.52) |  |
|  | Adult | 18 | 1 (5.5) | 0.14 - 27.29 | 1 (0.05 - 17.32) |  |
| Sex | Male | 56 | 3 (5.35) | 1.11 - 14.86 | Reference | 0.819 |
|  | Female | 24 | 1 (4.16) | 0.10 - 21.12 | 0.76 (0.07 - 7.78) |  |
| Status of  Health | Healthy | 16 | 0 | - | 1 (omitted) | 0.305 |
|  | Sick | 64 | 4 (6.25) | 1.72 - 15.23 | Reference |  |
| Use of  Disinfectant | Yes | 73 | 4 (5.47) | 1.51 - 13.4 | 1 (omitted) | 0.525 |
|  | No | 7 | 0 | - | Reference |  |
| Dermatitis | Found | 2 | 0 | - | 1 (omitted) | 0.742 |
|  | Not Found | 78 | 4 (5.12) | 1.41 - 12.61 | Reference |  |
| Wound | Yes | 4 | 0 | - | 1 (omitted) | 0.638 |
|  | No | 76 | 4 (5.26) | 1.45 - 12.93 | Reference |  |
| Otitis externa | Yes | 5 | 0 | - | 1 (omitted) | 0.596 |
|  | No | 75 | 4 (5.33) | 1.4 - 13.09 | Reference |  |
| Shower  interval | Regular | 16 | 1 (6.25) | 0.15 - 30.23 | 1.35 (0.13 - 13.96) | 0.802 |
|  | Irregular | 64 | 3 (4.68) | 0.97 - 13.09 | Reference |  |
| Vaccination | Yes | 40 | 1 (2.5) | 0.06 - 13.15 | 0.31 (0.03 - 3.17) | 0.294 |
|  | No | 40 | 3 (7.5) | 1.57 - 20.38 | Reference |  |
| Deworming | Yes | 44 | 1 (2.27) | 0.05 - 12.02 | 0.25 (0.02 - 2.57) | 0.211 |
|  | No | 36 | 3 (8.33) | 1.75 - 22.46 | Reference |  |
| Previous use of antibiotics | Yes | 45 | 3 (6.6) | 1.4 - 18.26 | 2.42 (0.24 - 24.41) | 0.438 |
|  | No | 35 | 1 (2.8) | 0.07 - 14.91 | Reference |  |
| Present use of  antibiotics | Yes | 17 | 0 | - | 1 (omitted) | 0.286 |
|  | No | 63 | 4 (6.34) | 1.75 - 15.46 | Reference |  |
| Use of Steroid | Yes | 5 | 0 | - | 1 (omitted) | 0.596 |
|  | No | 75 | 4 (5.33) | 1.4 - 13 | Reference |  |
| Use of Topical  Cream | Yes | 6 | 0 | 0 - 45.92 | 1 (omitted) | 0.559 |
|  | No | 74 | 4 (5.40) | 1.49 - 13.26 | Reference |  |
